# Encoding contact size using static and dynamic electrotactile finger stimulation: natural decoding vs. trained cues

**DOI:** 10.1007/s00221-024-06794-y

**Published:** 2024-03-12

**Authors:** Mauricio Carlos Henrich, Martin A. Garenfeld, Jovana Malesevic, Matija Strbac, Strahinja Dosen

**Affiliations:** 1 Department of Health Science and Technology, Aalborg University, Selma Lagerløfs Vej 249, 9260 Gistrup, Denmark; 2 Tecnalia Serbia Ltd, Deligradska 9/39, 11000 Belgrade, Serbia

**Keywords:** Electrotactile stimulation, Size perception, Tactile feedback, Virtual reality, Haptic device

## Abstract

Electrotactile stimulation through matrix electrodes is a promising technology to restore high-resolution tactile feedback in extended reality applications. One of the fundamental tactile effects that should be simulated is the change in the size of the contact between the finger and a virtual object. The present study investigated how participants perceive the increase of stimulation area when stimulating the index finger using static or dynamic (moving) stimuli produced by activating 1 to 6 electrode pads. To assess the ability to interpret the stimulation from the natural cues (natural decoding), without any prior training, the participants were instructed to draw the size of the stimulated area and identify the size difference when comparing two consecutive stimulations. To investigate if other “non-natural” cues can improve the size estimation, the participants were asked to enumerate the number of active pads following a training protocol. The results demonstrated that participants could perceive the change in size without prior training (e.g., the estimated area correlated with the stimulated area, *p* < 0.001; ≥ two-pad difference recognized with > 80% success rate). However, natural decoding was also challenging, as the response area changed gradually and sometimes in complex patterns when increasing the number of active pads (e.g., four extra pads needed for the statistically significant difference). Nevertheless, by training the participants to utilize additional cues the limitations of natural perception could be compensated. After the training, the mismatch in the activated and estimated number of pads was less than one pad regardless of the stimulus size. Finally, introducing the movement of the stimulus substantially improved discrimination (e.g., 100% median success rate to recognize ≥ one-pad difference). The present study, therefore, provides insights into stimulation size perception, and practical guidelines on how to modulate pad activation to change the perceived size in static and dynamic scenarios.

**Supplementary Information:**

The online version contains supplementary material available at 10.1007/s00221-024-06794-y.

## Introduction

In virtual and augmented reality (VR/AR) applications, the user can visually and audibly interact with the simulated environment, which thereby creates an immersive feeling. The illusion of physical presence is, however, prone to be broken when trying to touch virtual objects as commercial technologies do not provide haptic effects (Wang et al. 2019). Restoring haptic feedback is equally important in telemanipulation scenarios, where the operator uses a remote robot to interact with the environment (e.g., grasp and manipulate a remote object) (Lin et al. 2022; Park et al. 2022). The human hands are dexterous sensory organs and are instrumental for object manipulation and the exploration of the surroundings. A dense population of skin mechanoreceptors encodes information regarding the physical properties of an external stimulus into a train of action potentials that are conducted by the somatosensory system to the brain. To maintain the feeling of immersion and potentially improve user experience as well as performance in VR/AR and telerobotic applications, the physical properties of the external stimulus must be therefore encoded via haptic feedback (Johansson and Flanagan 2009). A simple encoding that allows such properties to be naturally perceived by the user through artificial feedback is critical for making the virtual and remote experience closer to the natural interaction (Preusche and Hirzinger 2007).

Previous clinical and industrial solutions have attempted to convey tactile and kinesthetic feedback using different technologies. Kinesthetic solutions simulate mechanical interaction by applying resistive forces to the hand/fingers using mounted exoskeletons (Choi et al. 2016; CyberGrasp; In et al. 2011) or stationary desktop devices e.g., Omega (omega.3) and Touch haptic device (Touch haptic device). Tactile feedback, on the other side, can be provided using various technologies to stimulate the skin such as smart materials (piezoelectric actuators, electroactive polymers, pneumatic actuators) (Xie et al. 2017), ultrasound, pin-arrays, skin-stretch, and vibrotactile stimulation (Bermejo and Hui 2021; Pacchierotti et al. 2017). Although kinesthetic solutions allow communicating modality-matched feedback (force reflected as force), a common drawback of those technologies is that they are bulky and therefore challenging to translate into a compact wearable system. Vibrotactile actuators can be rather small and have been embedded into a glove (Gollner et al. 2012); however, sound from the motors and mechanically-imposed coupling between the parameters are the limiting factors (Azadi and Jones 2014).

Electrotactile stimulation is another approach to providing tactile feedback using a compact solution, high spatial resolution, and flexible stimulation profiles thanks to the independent modulation of stimulation parameters (frequency, amplitude and pulse width) (Kourtesis et al. 2021; Zhou et al. 2022a, b). To elicit specific sensations, a fundamental understanding of skin structure, which serves as a complex interface for sensory perception, is crucial. Within the human skin, there are four distinct types of mechanoreceptors, each tuned to detect specific skin deformations. Electrical stimulation is by nature an artificial stimulus and therefore the quality of the evoked sensation is different than those produced by natural stimuli (such as mechanical stimulation). While the latter naturally activates one or more of sensory receptors, depending on the characteristics of the stimulus (e.g., low vs. high frequency, push vs. stretch), an electrical pulse directly and non-selectively depolarizes the underlying afferent fibers. The electrical stimulation therefore bypasses the mechanical transduction, and if the stimulation parameters are not carefully adjusted, it can produce a sensation that might not feel natural (so called “electrical” touch). The quality of electrotactile sensations was investigated extensively in the literature (Dong et al. 2020; Geng et al. 2018; Zhou et al. 2022a, b). There is also a risk of referred or spreading sensations when the stimulation activates afferent fibers innervating distal skin segments. In this case, the elicited sensation may extend beyond the targeted area, further complicating the reliable recreation of tactile perceptions.

In addition to independent and fine modulation of pulse parameters, another advantage of electrotactile stimulation is that the electrodes can be produced with different number and arrangement of stimulation pads. The conductive electrode pads can be printed on slim materials (Isakovic et al. 2022; Maleševic et al. 2021; Štrbac et al. 2016) and small inter-pad distance enables a spatial resolution that is only limited by the two-point discrimination (Solomonow et al. 1977, 1978). These characteristics allow using electrotactile stimulation to approximate some features of the natural feedback, for instance, high fidelity and spatial distribution. An illustrative example that demonstrates the capabilities of electrotactile technology is the stimulation of the fingertip using multi-channel electrodes (Bobich et al. 2007; Hummel et al. 2016; Isakovic et al. 2022; Ishizuka et al. 2017; Kaczmarek et al. 1997; Kaczmarek and Haase 2003; Kajimoto et al. 2004; Maleševic et al. 2021; Warren et al. 2008; Yem and Kajimoto 2017). In this approach, multiple pads are placed over the fingertip and the results reported in the literature demonstrate that participants can discriminate individual electrode pads (Bobich et al. 2007; Isakovic et al. 2022; Warren et al. 2008), recognize spatial patterns (Hummel et al. 2016; Kaczmarek and Haase 2003; Kajimoto et al. 2004), and parameters of the electrical stimulus (intensity and frequency) (Hummel et al. 2016; Ishizuka et al. 2017), despite a rather confined space over which the stimulation is applied (tip of the finger).

Ideally, the provided haptic feedback should convey all aspects of tactile interaction between the hand and object, and the point of contact is one of the most basic effects (Hummel et al. 2016; Yem and Kajimoto 2017) (e.g., the sensation indicating that the object touched the tip, lateral or medial side of the fingertip). Importantly, during grasping, the area through which the human hand establishes contact with an object can vary in size, and this can also change during the interaction. For instance, when pressing a finger into a compliant object with an increasing force, the object surface “wraps” increasingly around the fingertip (Bicchi et al. 2000; Fujita and Ohmori 2001; Takei et al. 2015). The contact area is encoded by the sensory system through multiple sensory integrative mechanisms. The increasing contact surface and possibly pressure will invade into receptive fields of more mechanoreceptors whose neural fibers will fire at increasing frequencies. The spatial summation in the convergent central nervous system plays a role as early as in the spinal neurons and is integrated into a larger network when it reaches the cortical level for conscious perception (Johnson et al. 2000).

Importantly, both of these natural mechanisms, namely, rate and spatial encoding, can be mimicked by the electrotactile stimulation through the modulation of pulse frequency and change in pad state (on/off), respectively. For instance, the area of stimulation can be expanded by sequentially activating more and more pads of the electrode, around the pad corresponding to the point of initial contact. As shown in (Hummel et al. 2016; Kajimoto et al. 2001), the interaction force can be communicated by modulating the intensity or frequency of stimulation, but it could be also conveyed by expanding the stimulated area to simulate the increase in the size of the contact, as explained in the previous example (e.g., pressing into a compliant object). Modulating the contact area could represent additional tactile effects such as an object slipping in or out of the hand, or gradual immersion in liquid (Ferguson et al. 2018). Therefore, communicating the size of the contact in static and dynamic scenarios is indeed an important effect to consider when designing tactile feedback systems.

In the present study, we investigated if and how well the high-resolution electrotactile stimulation delivered to the fingertip through a matrix electrode can be used to convey the size of the contact. More specifically, we assessed the quality of contact size estimation when the participants used natural decoding, without any prior information on the delivered stimulation patterns, as well as after they received a brief training. We have also considered whether the movement of the electrotactile stimulus affects the contact size perception. As explained above, electrotactile stimulation can produce a complex response, which includes substantial spreading and referred sensations, and this can have a substantial impact on the size and shape of the evoked percepts. Therefore, we assumed that natural decoding is feasible but also limited (in resolution, as well as size and/or shape estimation), and that those limitations can be overcome by a short training. To assess the natural decoding, the participants were asked to a) draw on a virtual finger the perceived sensation elicited by activating a different number of pads within a matrix electrode; and b) identify the “larger” stimuli from a train of 2 sequential stimuli of varying sizes (relative size estimation). To assess the effect of training, the participants performed the numerosity test where they identified the number of activated pads (absolute size estimation) before and after brief training.

## Methods

### Participants

Ten healthy volunteers (9 males and 1 female, 28.5 ± 7 yrs.) participated in the experiment. The participants were informed about the aim and purpose of the study, and informed consent was obtained before the start of the experiment. The study received approval from the Ethical Committee of the Region Nordjylland, Denmark (VN: 20150075).

### Electrotactile stimulation

**Fig. 1:**
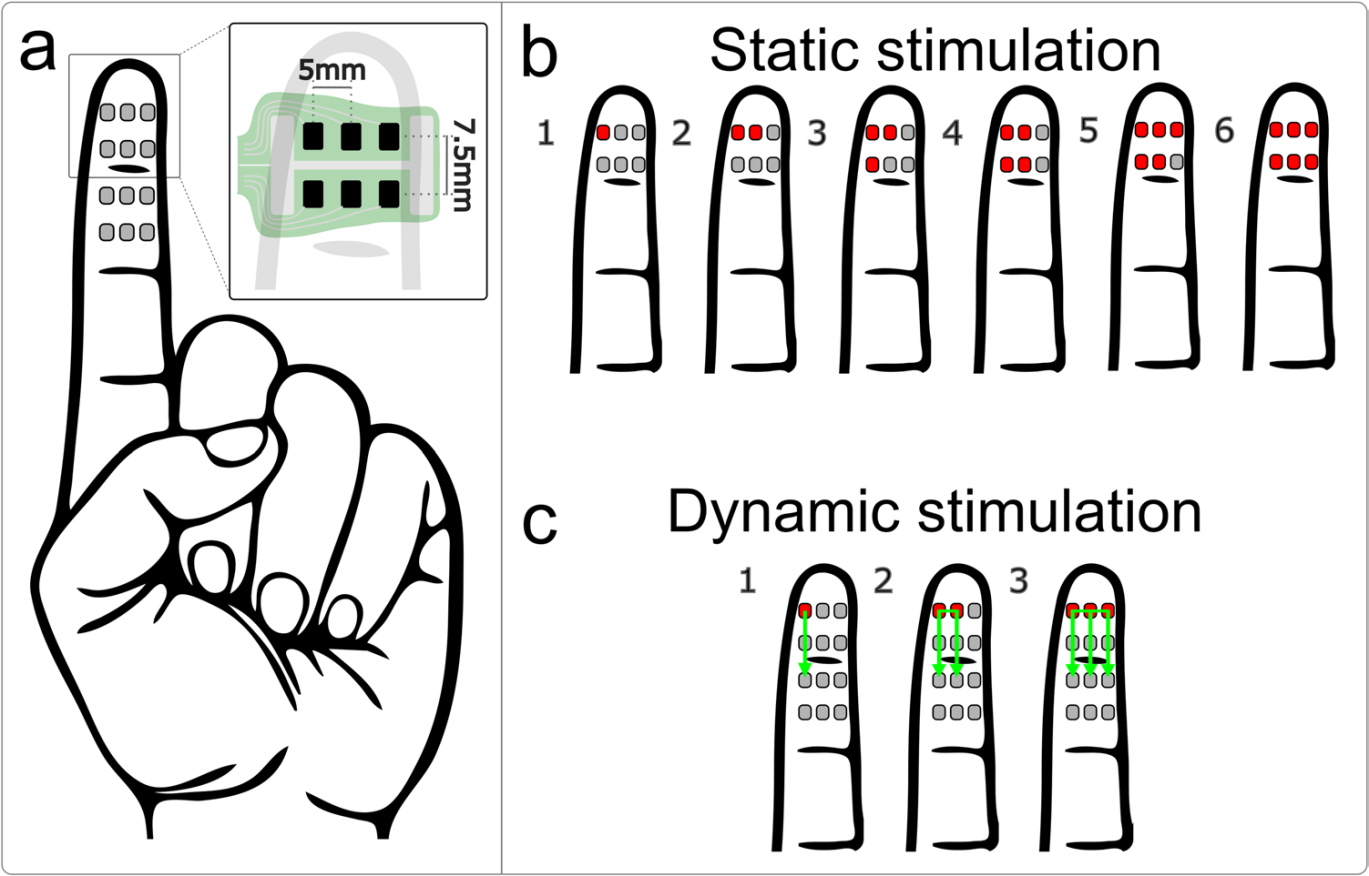
Electrotactile stimulation was delivered to the index finger of the left hand, as a static or dynamic stimulation pattern. Two matrix electrodes were placed on the middle phalange and the fingertip as showed in (**a**). Static stimuli included six configurations activating one to six pads simultaneously, see (**b**). Dynamic stimulation was delivered by sequentially activating the pads in the distal to proximal direction (visualized by the green arrows) simulating straight lines of varying widths (one, two, or three pad columns), see (**c**). The active pads are shown in intense red, while the grey color indicates inactive pads.

Electrotactile stimulation was delivered to the index finger of the non-dominant hand using a constant-current stimulator (Tactility Gamma, Tecnalia Research and Innovation, Spain) through a multi-array electrode for the stimulation of fingertips developed by screen-printing of medical grade conductive and dielectric materials on a PET-based flexible foil substrate (Tactility v2.1, Tecnalia Serbia). The stimulator is an improved version of the device that has already been used to deliver electrotactile stimulation to the index finger (Vizcay et al. 2023). The non-dominant hand was selected as the target for the stimulation so that the participants could use their dominant hand in the drawing test (as explained later). Two identical electrodes were positioned on the distal and middle phalange of the index finger. Each electrode integrated a 2 × 3 square arrangement of active pads enclosed by an H-shaped reference pad (Fig. 1a). The stimulation was delivered as a train of symmetric biphasic rectangular pulses and could be modulated in amplitude, pulse width, and frequency in the range of 500–9000 µA, 30–500 µs, and 1–200 Hz and in steps of 100 µA, 10 µs, and 1 Hz, respectively. Since the active pad was considerably smaller compared to the reference pad, the stimulus was perceived mostly under the active pads.

To calibrate the stimulation, the sensation threshold (ST) was estimated for each active pad using the ascending method of limits, starting from 500 μA and increasing in steps of 100 μA per second. The pulse width and frequency were kept constant at 400 μs and 30 Hz, respectively, throughout the entire experimental session. The pulse amplitude was set to 1.2 × ST (Maleševic et al. 2021), and if required, fine-tuned to produce a clear, fused, and localized sensation with similar intensity across pads. Importantly, the aim was to generate comfortable sensation that can be perceived by the participant. Adjusting the quality of elicited percepts to be as close as possible to natural touch would require more effort and was outside the scope of the present study but was addressed in the literature (Dong et al. 2020; Geng et al. 2018).

### Experimental protocol

The participants were comfortably seated in a chair in front of a computer screen where the instructions were displayed. The stimuli were delivered to the non-dominant hand, which they were asked to keep in a resting position on a padded surface on the table in front of them. The skin on the volar side of the index finger was cleaned with an alcohol wipe (70%), and the electrodes were placed on the two distal phalanges of the index finger. The medical tape was used to fixate the electrode to the skin, ensuring good skin–electrode contact. Precautions were taken not to tighten the tape too much to avoid eliciting the perception of blood pulsing in the finger, as this might confound the perception of stimuli delivered to the finger. The STs were obtained as described above and the participants were then familiarized with the electrotactile stimulation by delivering a set of stimulation patterns (three repetitions of each condition: Fig. 1b) in random order.

**Fig. 2:**
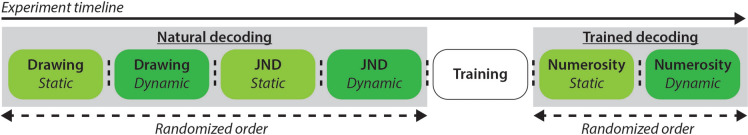
Schematic illustration of the sequence of events during the experimental session. The dashed vertical lines indicate breaks between the tests. The JND and Drawing test assess natural decoding (before training) whereas the Numerosity assessment is conducted after the training

Six different tests were performed to assess how the participants perceived the size of the electrotactile stimulus when the stimulation was delivered statically and dynamically. In the static approach, a set of pads forming the desired pattern was activated simultaneously and continuously for the duration of the stimuli. In the dynamic paradigm, the desired pattern was “moved” across the finger by activating neighboring pads in the same arrangement (Fig. 1c). The experimental session was therefore divided into two blocks, static and dynamic assessment, and three tests (drawing, JND, and numerosity) were performed in each block (Fig. 2). The order of the blocks and the tests within the blocks were randomized, except for the numerosity tests that were always performed last. The drawing and JND tests aimed to assess the natural perception without any prior training that could lead to biased associations between stimulus and perception (i.e., natural decoding of the electrotactile stimulus). As the numerosity tests included a training phase, it was administrated at the end of the session. As explained later, during the training, the electrotactile patterns were disclosed to the participants visually, so that they knew which patterns they could expect to receive and could associate the pattern to the characteristics of the elicited sensation.

#### Drawing tests

The participants were asked to mark the perceived stimulated area over a virtual hand shown on the screen. This test aimed to assess if and how well the spatial extent of the perceived sensation would correlate with the physical size of the electrotactile stimulus defined by the number of pads activated during stimulation. For the static testing, the stimulation patterns were designed to mimic an increase in the size of the contact, e.g., when grasping an object with increasing forces. Six patterns were used, in which different numbers of pads were activated simultaneously to simulate gradual spread in the area of contact starting from the upper left corner of the fingertip (see Fig. 1b). The stimulus duration for each pattern was 1.5 s. The participants received five repetitions of each stimulus in random order (a total of 30 stimuli). After each stimulus, the virtual hand was shown on the screen in front of them, and they were instructed to draw using a computer mouse the outline of the area where they perceived the stimulus. As usual (D’Anna et al. 2017; Fifer et al. 2022; Marasco et al. 2011; Shaballout et al. 2019; Tan et al. 2015), the size of the virtual hand was always the same (not scaled to the participant). Importantly, the participants could edit the drawing as much as they wanted to minimize the errors due to poor drawing skills. This step normally took from 5 to 10 s. The total area marked by the participant was quantified and compared between the stimuli. The quantification was performed by summing up the number of pixels within the virtual finger that were outlined by the participant in each trial. The “affected” area was then expressed as a percent of the total area of the finger. During the dynamic testing, the stimulation patterns also mimicked the contacts of different sizes (1, 2, and 3 pads; 5 repetitions of each condition, a total of 15 stimuli), but in this case, the contact area was moving by activating the pads sequentially along a straight line in the distal to proximal direction (see Fig. 1c). The stimulus duration of each sequence was 500 ms, resulting in 1.5 s for the total duration of the “line” stimulus (same as in the static condition). Importantly, during these tests, the participants did not receive any a priori information about the features of the stimulation patterns (i.e., number and arrangement of pads) that were delivered to them.

#### Just Noticeable Difference (JND) tests

This test was designed to estimate the minimum difference in the size of two stimuli that can be reliably perceived by the participants. While the drawing tests assessed if the spatial extent of the elicited sensation increased with the size of the electrotactile stimulus, the JND test investigated if that increase was large enough for the participant to consciously and reliably recognize that the two stimuli were of different sizes. To assess this during the static stimulation, two electrotactile stimuli were delivered sequentially, where the reference stimulus always included a single pad stimulation (pattern 1, in Fig. 1b), while the test stimulus comprised the simultaneous activation of 2–6 pads (patterns 2–6, in Fig. 1b), hence a difference of 1–5 pads between the two stimuli. Each of the test patterns was presented 5 times and the order of presentation was randomized (giving a total of 25 trials), to prevent response bias (Rohde et al. 2016). The duration of the two sequential stimuli was 1.5 s (as in the previous test) with a break of 1 s between them, and the reference and test stimulus were delivered in random order. The participants were then asked to indicate whether the sensation area elicited by the two stimuli was the same or different in size. The success rate in identifying that the stimuli were indeed of different sizes was calculated for all size differences between the reference and test stimuli (1–5 pads), and they were then compared statistically. Similarly, during the dynamic testing, the “lines” of different widths were delivered (see Fig. 1c), following the same protocol as in the static condition, and the participants were asked to indicate whether the area of the finger activated by the two stimuli was of the same or different size. As in the drawing test, the participants were not provided any information about the stimulation patterns that were delivered to them.

#### Numerosity tests

Lastly, the numerosity test assessed the participants’ ability to enumerate the number of active pads. The JND tests evaluated the relative size discrimination, whereas the present test, assessed if the participants could judge the absolute size, by recognizing the number of active pads. In addition, while the previous tests assessed the natural response to stimulation without any a priori knowledge about the stimulation patterns, in the present test, the participants were briefly trained to recognize the size of the stimuli. The rationale behind introducing the training phase was to support the association between a perceived sensation and a specific number of active pads.

First, a familiarization phase was conducted in which the stimulation patterns (Fig. 1b) were sequentially delivered to the participant in the order of increasing size. Each stimulus was delivered five times (a total of 30 stimuli), and during the stimulation, the patterns were also visually presented to the participant to associate tactile and visual information. In the reinforced learning phase, the same stimuli were presented randomly, this time without visual feedback, and the participant was asked to indicate the number of active pads (stimulus size). After the participants provided their answer, the correct number of pads was revealed; hence, allowing them to learn from their mistakes. Each stimulation pattern was presented five times (a total of 30 stimuli). Finally, in the validation phase, the protocol was the same as in the reinforced learning phase but no feedback about the correct number of activated pads was provided to the participant (a total of 30 stimuli). The duration of stimulation was 1.5 s as in the previous tests. The outcome of this test was the “size perception mismatch”, defined as the difference between the reported and the actual number of activated pads. The perception mismatch was quantified for each stimulus (# of pads) and compared between the stimuli. The dynamic version of the numerosity test was performed following the same protocol but using the dynamic “line” patterns composed of one, two, and three pads (Fig. 1c).

### Statistical analyses

The collected datasets were tested for normality using the Shapiro–Wilk test. Since deviations from normality were confirmed, non-parametric tests were applied for statistical comparisons. Friedman test was used to assess significant differences between the conditions. Tukey’s honestly significant difference (HSD) procedure was applied for multiple pairwise comparisons when a significant difference was detected. In addition, for the drawing tests, a repeated measures correlation analysis was performed to assess whether there was a linear correlation between the number of activated pads and the perceived stimulated area. A significance threshold of *p* < *0.05* was assumed. The results are reported separately for both Static and Dynamic tests. The data is presented in boxplots to show the distribution, and stimulation intensities are reported in the text as mean ± standard deviation. Data processing and statistical tests were performed using Matlab R2022b.

## Results

### Drawing tests

**Fig. 3:**
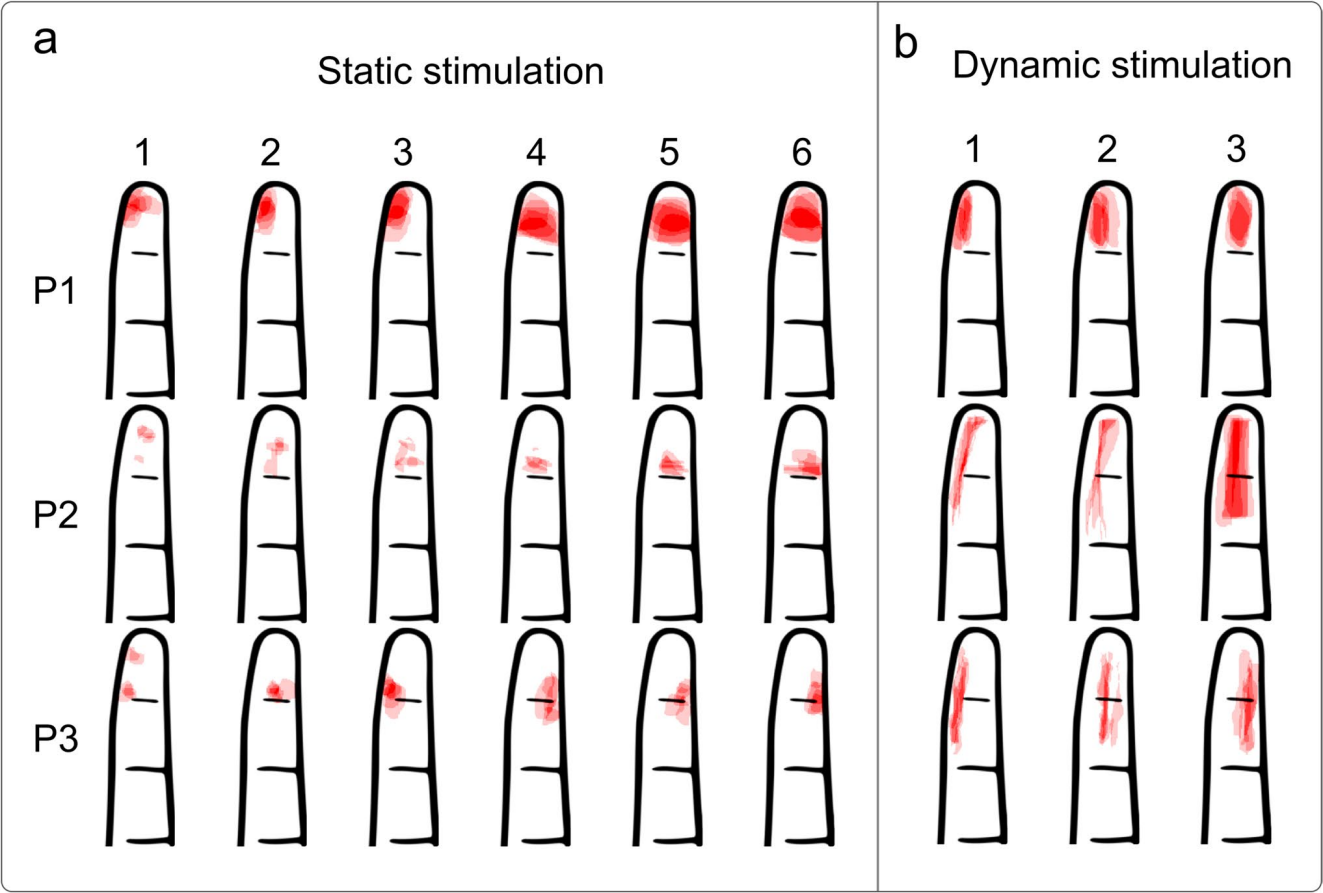
Representative drawings of the perceived area of sensation in three subjects in response to static (**a**) and dynamic stimulation (**b**). The areas marked by each subject in the five repetitions of each stimulus are normalized according to transparency to indicate the frequency of the marked area across trials. The numbering within each column corresponds to the stimulation patterns for static and dynamic stimulation as shown in Fig. 1b and c, respectively

The average stimulation amplitude (mean ± standard deviation) after calibration, over all pads and participants, was 2014 ± 1157µA. The drawings of three representative participants are shown in Fig.﻿ 3, for the static (left column) and dynamic stimulation (right column). The pixel values (i.e., 0 if not marked or 1 if marked) were added across trials and divided by the total number of pixels associated with the whole finger. The transparency of the color, therefore, indicates how often the specific pixels (areas) were marked for the given stimulation pattern. In general, the marked areas correlate with the size of the delivered stimulus (number of pads) in both static and dynamic conditions. However, the relation between the activated pads and elicited sensation seems to be complex and variable across participants. For instance, the increase in the size of the marked area can be visually appreciated only when there is a large difference in the size of the stimulation patterns, see static stimulation drawings. Furthermore, the marked areas do not necessarily correspond to the spatial arrangement of the activated pads, and this is particularly visible in static stimulation conditions (Fig. 3, P2 and P3), where the stimulation seems to be moving in position while also somewhat expanding. For P3, the shift (in addition to the spread) in sensation is also apparent for dynamic stimulation. The profiles obtained in other participants reflect those shown in Fig.﻿ 3 and can be seen in Supplemental Material, Fig. 1.

The repeated measures correlation analysis revealed that the size of the area marked by the participants on the virtual finger in response to the stimulation increased with the number of activated pads (see Fig.﻿ 4). The repeated measures correlation analyses showed a significant positive correlation between the number of activated pads and the perceived area of the elicited sensation, for both, static (Fig.﻿ 4) and dynamic stimulation (not shown).

**Fig. 4:**
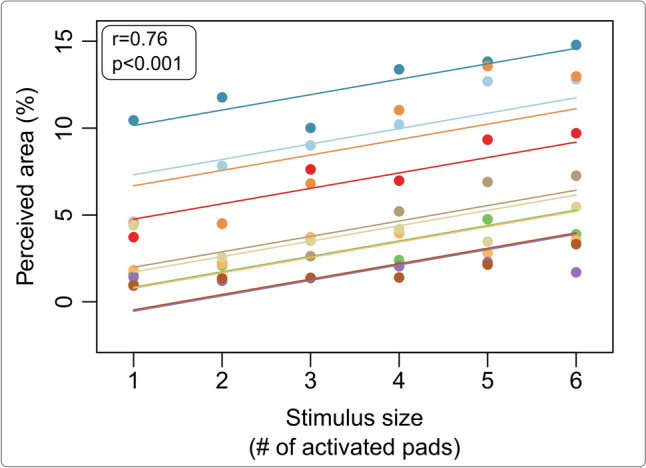
Repeated measures correlation analyses (Bakdash and Marusich 2017) showed an increase in the perceived stimulated area with the number of activated pads for static stimuli (stimulus size). The perceived stimulated area is expressed in percentage relative to the total area of the finger. The dots are the individual participants’ responses, and the full lines are the fitted linear regressions. The *r* and *p* values reported in the top left corner belong to the repeated measures correlation analysis that was performed to assess the relationship between the number of activated pads and the perceived stimulated area

**Fig. 5:**
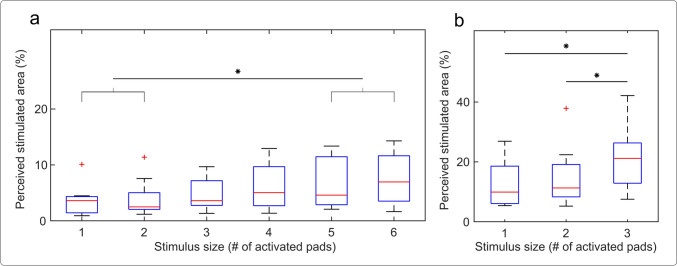
Box plots showing the area of perceived stimulation (vertical axis) marked in response to the number of active pads (horizontal axis), where (**a**) and (**b**) are static and dynamic stimulation, respectively. Perceived areas are expressed as the percentages of the total area of the finger. The red lines, blue boxes, black whiskers, and red crosses indicate the median, IQR, upper boundaries, and outliers, respectively. The asterisks show significant differences (*: *p* < 0.05)

This was confirmed also by the Friedman test (Fig. 5a, b), which indicated a significant effect of the number of pads on the perceived area (Friedman test, *p* < 0.001). In the post hoc tests for the static patterns, significant differences were detected for 1 vs. 5 and 6 pads, and 2 vs. 5 and 6 pads (HSD, *p* < *0.05*), confirming thereby a rather gradual increase in the area of the perceived sensations. Similarly, in the dynamic tests, the area associated with one- and two-pad lines was significantly smaller compared to 3-pad line (HSD, *p* < *0.05*).

### JND tests

**Fig. 6:**
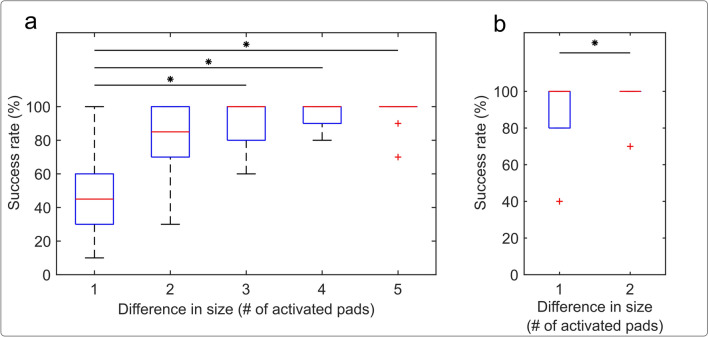
Box plots of the success rate in discriminating the difference in the stimulus size depending on the difference in the number of active pads between two sequential stimuli, where (a) and (b) are static and dynamic stimulation, respectively. Box plot information and significant differences are indicated as in Fig. 5

Figure 6 shows the success rate in discriminating the difference in the perceived size of the two sequential stimuli depending on the difference in the number of active pads in each stimulus. For static stimulation, a significant effect of the difference in the number of pads was found (Fig. 6a. Friedman test, *p* < 0.001). Post-hoc tests revealed a significantly lower success rate when the sequential stimuli differed by only a single pad vs. 3, 4, and 5-pad difference. The median success rates in the case of 2, 3, 4, and 5-pad differences were all higher than 80%. Considering that the JND is often defined statistically, as the difference that can be perceived in 75% of the cases (Kingdom and Prins 2009), the difference of 2 and more pads is equal to or higher than the JND.

Interestingly, the median success rate for the JND test in the dynamic condition (Fig. 6b) was 100% already for one-pad difference and the same for two-pad difference, although the two cases were characterized with different dispersions (see the IQRs). The significant difference, *p* < *0.05,* between the two conditions is most likely due to the larger variability of the responses when the line width differed by a single pad.

### The assessment of numerosity

**Fig. 7:**
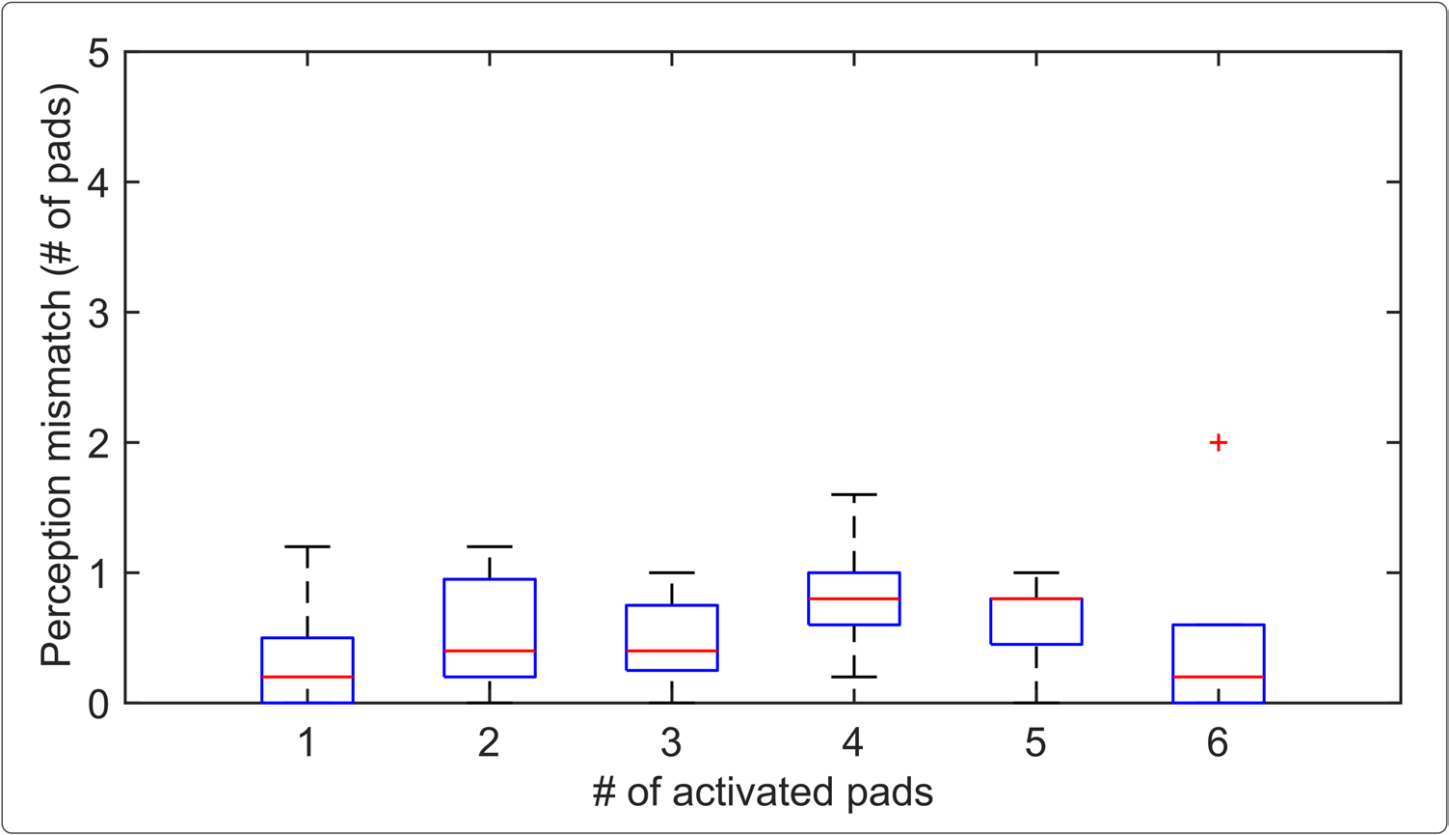
Box plot of the size perception mismatch (difference between the estimated and correct number of active pads) from the numerosity test for static stimulation. For better readability, results from the dynamic test are not reported as the median was zero in all trials. Box plot information is indicated as in Fig. 5. No significant differences were found between the conditions (Friedman test)

The summary results for the size perception mismatch as a function of the number of active pads in the stimulation pattern are shown in Fig. 7. In the static test, the median perception mismatch was below one pad regardless of the number of activated pads (1–6), with no significant difference across the number of pads (Friedman test). In the dynamic test, the median perception mismatch was equal to zero, suggesting that the participants could easily identify the exact size of the stimuli (one, two, and three pads) in this condition (Friedman test).

## Discussion

The present study investigated the capabilities of a matrix stimulation interface to convey spatial cues indicating the changes in the contact size, which is one of the fundamental haptic effects characterizing mechanical interaction (e.g., a finger pushing with an increasing force into a compliant object). Flexible matrix electrodes were placed on the distal phalanges of the index finger, and a series of static and dynamic tests were performed. The obtained results provide novel insights that can guide the implementation of an electrotactile interface in any application where high-density tactile feedback is used to simulate a realistic hand-object interaction (e.g., VR/AR and telemanipulation).

The results of the drawing tests demonstrated that activating more pads of the electrode produced tactile sensations that expanded over an increasing area of the finger. Therefore, this test suggested that the information on the spatial extent of an external stimulus (e.g., contact size, force) could be, at least in principle, encoded “naturally” by modulating the number of simultaneously activated pads. However, the increase in the area of the elicited sensations was rather gradual across the number of active pads, and the shape of the tactile response was variable and, in some cases, not clearly associated with the spatial location of the activated pads. For instance, although in most cases of the static stimulation test the sensation was confined to one “connected” area, sometimes the perceived area comprised two or more separated segments (Fig. 2, static stimulation, P2, and P3). As can be seen for the dynamic stimulation, the produced tactile sensation varied in size and location. However, in all cases, the perception was limited to a straight line between the two most distal phalanges. Therefore, the relation between the spatial structure of the electrotactile stimulus, defined by the number and position of the active pads, and that of the elicited response area is not always straightforward, but participant-specific and complex. This can reflect the fact that the electrotactile stimulation does not activate mechanoreceptors directly but acts non-specifically by recruiting the underlying nerve fibers. Exact recruitment depends on multiple factors, such as individual innervation patterns and finger anatomy, and this can give rise to a complex and variable response. Interestingly, such variability exists despite the stimulated area being small and highly sensitive (fingertip), but these results are in line with a recent study from our group (Isakovic et al. 2022), where similar complex responses have been registered (although in a different context). This can be an important factor to consider when choosing electrotactile stimulation to provide feedback, especially when the feedback shall be interpreted naturally and without any prior training. In this context, mechanical stimulation can be considered a preferred choice when the feedback should be interpreted naturally, when more consistent responses are required, and when prior training is not possible. Nevertheless, as noted in the Introduction, an electrotactile interface can provide high-fidelity feedback via a flexible and thin matrix electrode and compact electronics form factor with significantly reduced power consumption.

Indeed, the JND tests confirmed the results of the drawing assessment, especially regarding the gradual modulation of the response area. As expected, increasing the difference in size (number of active pads) between the test and reference stimuli facilitated their differentiation. More specifically, the test showed that adding a single pad to the electrotactile pattern was not enough for the participant to detect the change in the spatial extent of the electrotactile stimulus. The separation between the electrode pads in the present study (Fig. 1a) was within the range of the two-point discrimination threshold (2PDT) for electrotactile stimulation (3–7 mm Dargahi and Najarian 2004; Kaczmarek et al. 1991; Marcus and Fuglevand 2009; Van Boven and Johnson 1994)). However, this cannot fully explain the aforementioned results as we did not ask the participants to detect two separate stimulation points (as in 2PDT) but an increase in the size of the elicited sensation (which could still be perceived as a single “fused” stimulus). Therefore, to robustly encode a change in the size of the contact in the static condition without prior training, at least two additional pads should be activated. The JND test revealed another interesting insight, namely, that introducing the movement of the stimuli can substantially increase the participants’ ability to discriminate the size. While the one-pad difference in the static condition was practically indistinguishable, the same difference was almost perfectly discriminated in the dynamic condition (see Fig. 6b). These results support the conclusion that dynamic stimulation improves the ability of the participant to localize and recognize spatial aspects of certain somatosensory stimuli (Dargahi and Najarian 2004; Kaczmarek et al. 1991), most likely supported by quickly adapting fibers, as suggested in early studies of the moving two-point discrimination (Dellon 1978; Louis et al. 1984). However, it should also be considered that in the tests performed, the reference stimulus always included one pad, and the performance is likely to depend on the baseline number of pads (e.g., 5 vs. 6 pads likely to be more challenging to discriminate compared to 1 vs. 2 pads).

Since the integration of electrical stimuli at the peripheral and central level exploits both temporal and spatial cues (Henrich et al. 2021; Mørch et al. 2010), one might speculate that since dynamic patterns convey additional temporal information (compared to a static stimulus), the discrimination and localization of the stimulus are to be improved. Already in 1990, it was reported that our ability to detect changes in intensity is enhanced when there is a changing stimulus over a stable background (Gescheider et al. 1990). In addition, although efforts were made to calibrate the intensity of each pad to the same perceived intensity, the sequential stimulation during the dynamic test also carries changes in absolute amplitude over time. As previously reported, these transient changes in amplitude can create distinct sensations and thereby enrich perception (Gescheider et al. 1990; Gunther and O’Modhrain 2003). These experiments seem to support the idea that dynamic stimulation enriches perception. The exact mechanisms are still under debate and most likely include several integrative mechanisms along the neural axis. For instance, a mechanism of lateral inhibition might be facilitated when dynamically stimulating a small area of the skin: a stimulus producing an area of excitation surrounded by one of inhibition has been already demonstrated in many sensory organs (von Békésy 1967). This facilitated inhibition would reduce the size of the receptive fields of primary sensory fibers (and/or receptive fields of converging central neurons) (Bremner and Fitzgerald 2008), explaining the improvement in the discrimination accuracy of dynamic (vs. static) stimuli. Spatial integration has been extensively studied in the somatosensory system and it was shown to enrich perception when multiple stimuli are applied to the skin of healthy participants (Defrin et al. 2009). Mechanisms of spatial summation and lateral inhibition most likely play a role at peripheral, and central levels, integrating sensory information and shaping perception and behavioral responses (Badde and Heed 2016).

Finally, the numerosity tests were conducted to assess if the participants could recognize the exact number of activated pads and the width of a line (rather than the difference between the two stimuli). Since in this test, the participants were asked to report on the number of activated pads, the stimulation patterns were disclosed to them, and they received brief but systematic training. In this case, therefore, the participants could learn to associate not only the perceived area of the elicited sensation but also other cues (e.g., changes in intensity, quality, and/or shape of the felt sensation) to the specific stimulation pattern (number of pads), as also anecdotally noted in (Maleševic et al. 2021). The results showed that the participants could successfully recognize the size of a stimulus with a median error of less than one pad. Importantly, this result was consistent regardless of the number of pads, i.e., they could identify the size of a single pad equally well as the size of a six-pad stimulus.

More generally, the judgment of numerosity is a commonly used test in human psychophysics (Bergen and Julesz 1983). The numerosity has been extensively investigated in different senses, including touch using mechanical (Cohen and Henik 2016; Verlaers et al. 2014) and electrotactile stimulation (Nataletti et al. 2020). In the visual sense, previous studies have suggested that when the number of stimulation points is lower than four, participants can accurately and rapidly identify them (Katzin et al. 2019; Kaufman et al. 1949). The error rate and reaction time significantly increased when adding more stimuli (Katzin et al. 2019). In the somatosensory system, however, experiments using mechanical tactile stimulation showed that participants were able to subitize between two and six stimuli when they were delivered to different fingers (Cohen et al. 2014; Cohen and Henik 2016; Katzin et al. 2019; Riggs et al. 2006). As in the visual sense, when adding further stimuli, the performance declined. The present study is the first to report the results of a numerosity test using electrical stimulation within the most sensitive area of the finger. Therefore, after a brief training, complex electrotactile patterns within the fingertip can be used to convey multidimensional information to the participant. This is an encouraging result for the use of electrotactile stimulation not only for feedback but also for general-purpose communication (Jure et al. 2022) and hence further applications of these findings are to be investigated in future work.

In the present study, the duration of a single stimulus in all tests was set to 1.5 s based on previous studies (Garenfeld et al. 2023; Jure et al. 2022; Parsnejad et al. 2020) and confirmed in pilot tests, as the long enough duration to produce a sensation that can be clearly perceived by the participants. It is important to mention that this stimulus duration can likely be integrated by temporal summation. An increase in the perceived intensity can be the result of increasing stimulus duration or repetitions (Geng et al. 2012; Graczyk et al. 2016; Paredes et al. 2015). Even if temporal summation occurred for the individual stimuli in the present study, this would not impact the main conclusions since the stimulus duration was the same between compared conditions.

Overall, the results of the present study show that certain characteristics of the natural perception using electrotactile stimulation (size and direction) is generally correctly interpreted even without training, as the increased size of the stimulus leads to an increased area of response. However, the natural decoding of the tactile sensation has some limitations, as the response is gradual (below JND), complex, and irregularly shaped, as well as participant-specific. Therefore, the natural decoding of the static stimulus without any participant training might not be an optimal choice when implementing effective tactile feedback. However, the limitations of the natural perception can be compensated by a brief training that allows additional cues to be incorporated to improve the overall discrimination ability. In addition, in an actual application, the tactile stimulation will be supplemented with visual observation, and the fusion of the two sensory inputs might improve the overall experience, especially during prolonged use. Finally, this limitation exists only for static stimuli, whereas introducing the movement of the stimulus substantially increases the ability to perceive the contact size and to discriminate small spatial differences, and hence dynamic stimuli can be naturally encoded.

### Study limitations

It is known that electrotactile stimulation is prone to habituation (Buma et al. 2007), but this was not investigated in the present study due to the already long experimental session. Nevertheless, the effects of adaptation are not expected to influence the results systematically since the presentation of electrotactile stimuli was fully randomized within each test. Moreover, it has been already demonstrated that the adaptation can be significantly reduced by presenting the stimuli in an intermittent fashion (as in the present study) (Buma et al. 2007). Finally, the participants did not complain about the loss of sensitivity across the session.

The number of repetitions of the same stimulus in each test had to be limited to a few presentations to avoid excessive session duration. More repetitions could provide additional analysis, for instance, the fitting of the psychometric functions to the data. Such insights could be obtained in future work by conducting dedicated studies focusing in depth on some of the performed tests. An open question about the drawing test is how well the participants can draw what they feel. Nevertheless, this approach is routinely used across studies (D’Anna et al. 2017; Fifer et al. 2022; Marasco et al. 2011; Shaballout et al. 2019; Tan et al. 2015), and we have made an effort to minimize subjective bias, as explained in Methods.

As stated in the Methods section, participant recruitment for this study was not restricted based on gender/sex, leading to the inclusion of 9 out of 10 male participants. It is important to acknowledge that gender/sex may likely influence electroactile localization/perception performance (Da Silva et al. 2014; Geng et al. 2015; Geng and Achuthan Paramanathan 2016). A subsequent investigation incorporating an assessment of gender/sex effects could provide insights into, for instance, potential differences in JND values between the groups.

Finally, the way we used electrotactile stimulation to elicit and modulate tactile sensations is still far from the intricate activation of afferent fibers that characterize natural touch (Saal and Bensmaia 2014). As explained in Introduction, this approach has some intrinsic limitations, but one way to increase naturalness could be to exploit the computational models to generate naturalistic firing patterns (which could be mapped to electrical pulses of the stimulator) (Saal et al. 2017). This point combined with the fast activation of fibers afforded by electrical stimulation can be important to improve the quality of tactile effects. This is, however, outside the scope of the present study and remains to be tested in future work.

### Supplementary Information

Below is the link to the electronic supplementary material.

Supplementary file1 (DOCX 45487 KB)

## Data Availability

The datasets generated during and/or analyzed during the current study are available from the corresponding author upon reasonable request.
