# Supplementary material for: Encoding contact size using static and dynamic electrotactile finger stimulation: natural decoding vs. trained cues
Source: Exp Brain Res. 2024 Mar 12;242(5):1047–60. doi: 10.1007/s00221-024-06794-y (PMC11078849; doi:10.1007/s00221-024-06794-y)
Supplement: Supplementary file 1 — Supplementary file1 (DOCX 45487 KB) [file 221_2024_6794_MOESM1_ESM.docx]

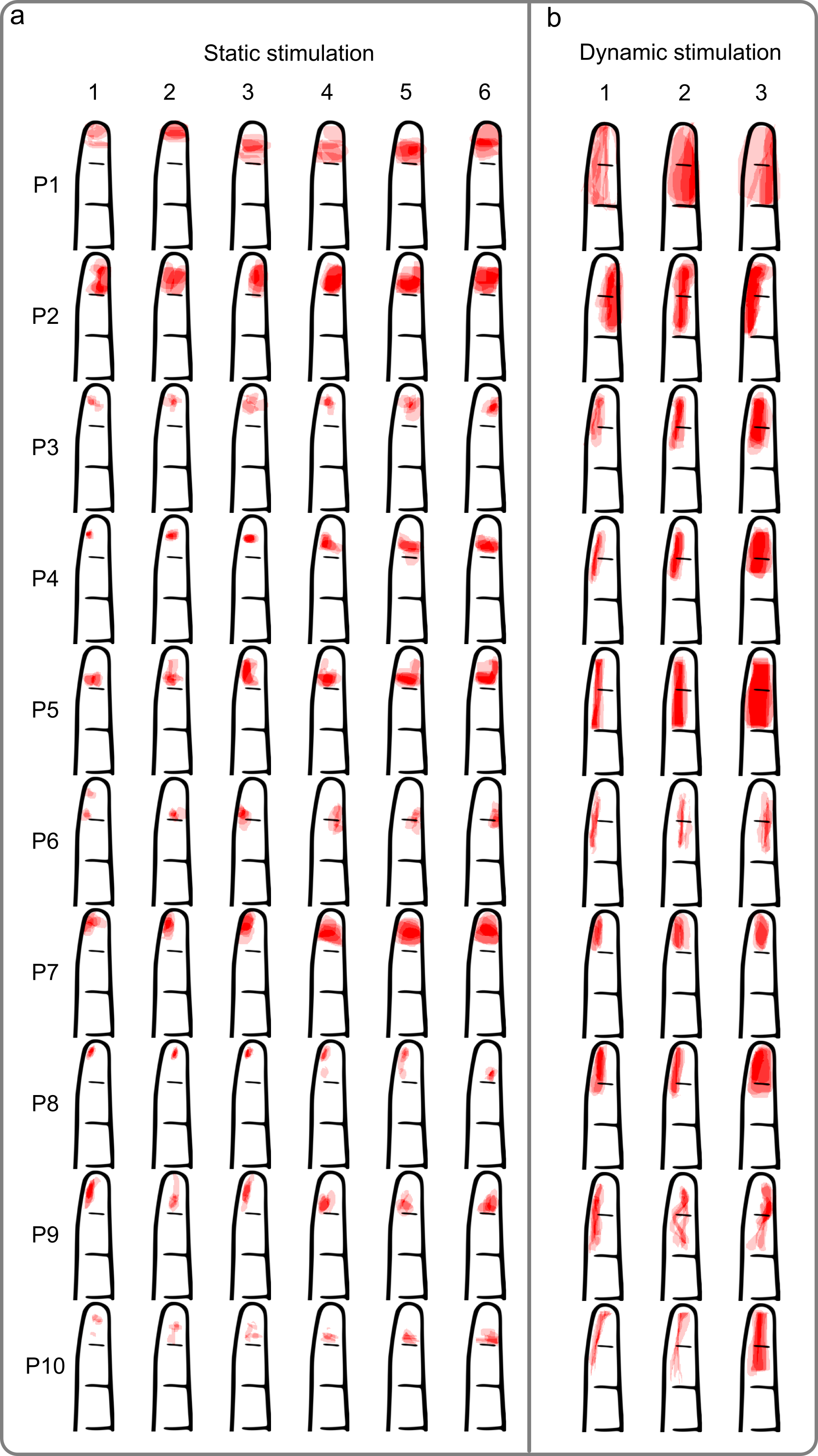


**Supplementary Fig. 1** Extension of *Fig. 3* showing drawings of the perceived area of sensation from all subjects. P1, P2 and P3 from *Fig. 3* correspond to P7, P10 and P6 in the present figure.

Article title: Encoding contact size using static and dynamic electrotactile finger stimulation: natural decoding versus trained cues

Journal name: Experimental Brain Research

Author names: Mauricio Carlos Henrich^1^, Martin A. Garenfeld,^1^ Jovana Malesevic^2^, Matija Strbac^2^ and Strahinja Dosen^1^

Affiliation: ^1^Department of Health Science and Technology, Aalborg University, Selma Lagerløfs Vej 249, 9260 Gistrup, Denmark and ^2^Tecnalia Serbia Ltd., Deligradska 9/39, 11000, Belgrade, Serbia.

E-mail address of the corresponding author: [sdosen@hst.aau.dk](mailto:sdosen@hst.aau.dk)
